# Supplementary material for: Recombinant SAG2A Protein from Toxoplasma gondii Modulates Immune Profile and Induces Metabolic Changes Associated with Reduced Tachyzoite Infection in Peritoneal Exudate Cells from Susceptible C57BL/6 Mice
Source: Microorganisms. 2024 Nov 20;12(11):2366. doi: 10.3390/microorganisms12112366 (PMC11596811; doi:10.3390/microorganisms12112366)
Supplement: Supplementary file 1 [file microorganisms-12-02366-s001.zip › microorganisms-3088826-supplementary.pdf]

microorganisms

MDPI

Supplementary Material

Recombinant SAG2A protein from *Toxoplasma gondii* modulates immune profile and induce metabolic changes associated with reduced tachyzoite infection in peritoneal exudate cells from susceptible C57BL/6 mice

Table S1. Exclusive or up-modulated proteins present in PECs from C57BL/6 mice treated with rSAG2A (FC >2 compared to PECs of BALB/c treated with rSAG2A).

QueryItem	Identity	Bitscore	PreferredName	Annotation
tr|A1BN54|A1BN54_MOUSE	98.0	1760.3	Actn1	Alpha-actinin-1; F-actin cross-linking protein which is thought to anchor actin to a variety of intracellular structures. This is a bundling protein (By similarity)
**tr|E9Q616|E9Q616_MOUSE**	**100.0**	**2275.4**	**Ahnak**	**AHNAK nucleoprotein (desmoyokin)**
**tr|Q6UL10|Q6UL10_MOUSE**	**96.9**	**768.8**	**Ahnak**	**AHNAK nucleoprotein (desmoyokin)**
tr|Q3UJW9|Q3UJW9_MOUSE	99.7	675.6	Akr1a1	Alcohol dehydrogenase [NADP(+)]; Catalyzes the NADPH-dependent reduction of a variety of aromatic and aliphatic aldehydes to their corresponding alcohols. Catalyzes the reduction of mevaldate to mevalonic acid and of glyceraldehyde to glycerol. Has broad substrate specificity.
sp|P24549|AL1A1_MOUSE	100.0	1023.1	Aldh1a1	Retinal dehydrogenase 1; Can convert/oxidize retinaldehyde to retinoic acid.
**tr|A0A2C9F2D2|A0A2C9F2D2_MOUSE**	**95.3**	**682.2**	**Anxa7**	**Annexin A7; Calcium/phospholipid-binding protein which promotes membrane fusion and is involved in exocytosis; Belongs to the annexin family**
tr|A0A0U1RPS0|A0A0U1RPS0_MOUSE	100.0	105.1	Ap2s1	AP-2 complex subunit sigma; Component of the adaptor protein complex 2 (AP-2). Adaptor protein complexes function in protein transport via Transport vesicles in different membrane traffic pathways.
**tr|Q99KA3|Q99KA3_MOUSE**	**100.0**	**810.1**	**Atp6v1b1**	**ATPase, H+ transporting, lysosomal V1 subunit B1**
**tr|Q3U0I3|Q3U0I3_MOUSE**	**100.0**	**995.3**	**Cct3**	**T-complex protein 1 subunit gamma; Molecular chaperone; assists the folding of proteins upon ATP hydrolysis. As part of the BBS/CCT complex may play a role in the assembly of BBSome, a complex involved in ciliogenesis regulating transports vesicles to the cilia.**
sp|Q04447|KCRB_MOUSE	100.0	787.7	Ckb	Creatine kinase B-type; Reversibly catalyzes the transfer of phosphate between ATP and various phosphogens (e.g. creatine phosphate). Creatine kinase isoenzymes play a central role in energy transduction in tissues with large, fluctuating energy demands, such as skeletal muscle, heart, brain and spermatozoa; Belongs to the ATP:guanido phosphotransferase Family
tr|Q3TF74|Q3TF74_MOUSE	99.8	968.4	Coro1c	Coronin-1C; May be involved in cytokinesis, motility, and signal transduction

tr|Q3TC17|Q3TC17_MOUSE	99.4	738.4	Ctsb	Cathepsin B; Thiol protease which is believed to participate in intracellular degradation and turnover of proteins. Has also been implicated in tumor invasion and metastasis; Belongs to the peptidase C1 family
tr|Q56A15|Q56A15_MOUSE	100	222.2	Gm10053	Predicted gene 10053
**tr|D3Z074|D3Z074_MOUSE**	**99.0**	**1694.9**	**Diap1**	**Protein diaphanous homolog 1; Acts in a Rho-dependent manner to recruit PFY1 to the membrane. Required for the assembly of F-actin structures, such as actin cables and stress fibers. Nucleates actin filaments. Required for cytokinesis, and transcriptional activation of the serum response factor.**
**tr|Q3TIE8|Q3TIE8_MOUSE**	**99.4**	**984.9**	**Dld**	**Dihydrolipoyl dehydrogenase, mitochondrial; Lipoamide dehydrogenase is a component of the glycine cleavage system as well as an E3 component of the alpha-ketoacid dehydrogenase complexes (pyruvate-, alpha-ketoglutarate-, and branched-chain amino acid-dehydrogenase complex).**
tr|A0A0R4J1E2|A0A0R4J1E2_MOUSE	100.0	1127.1	Eef1d	Elongation factor 1-delta; Isoform 1: EF-1-beta and EF-1-delta stimulate the exchange of GDP bound to EF-1-alpha to GTP, regenerating EF-1- alpha for another round of transfer of aminoacyl-tRNAs to the ribosome
tr|Q8C845|Q8C845_MOUSE	100.0	407.5	Efhd2	EF-hand domain-containing protein D2; May regulate B-cell receptor (BCR)-induced immature and primary B-cell apoptosis. Plays a role as negative regulator of the canonical NF-kappa-B-activating branch. Controls spontaneous apoptosis through the regulation of BCL2L1 abundance
tr|Q3TFG3|Q3TFG3_MOUSE	99.8	795.8	Eif4a1	Eukaryotic initiation factor 4A-I; ATP-dependent RNA helicase which is a subunit of the eIF4F complex involved in cap recognition and is required for mRNA binding to ribosome. In the current model of translation initiation, eIF4A unwinds RNA secondary structures in the 5'-UTR of mRNAs which is necessary to allow efficient binding of the small ribosomal subunit, and subsequent scanning for the initiator codon
tr|Q8C4S7|Q8C4S7_MOUSE	100.0	1111.3	Erap1	Endoplasmic reticulum aminopeptidase 1; Aminopeptidase that plays a central role in peptide trimming, a step required for the generation of most HLA class I- binding peptides. Peptide trimming is essential to customize longer precursor peptides to fit them to the correct length required for presentation on MHC class I molecules.
**sp|Q80X90|FLNB_MOUSE**	**100.0**	**5316.5**	**Flnb**	**Filamin-B; Connects cell membrane constituents to the actin cytoskeleton. Anchors various transmembrane proteins to the actin cytoskeleton (By similarity)**
tr|Q3U0C4|Q3U0C4_MOUSE	99.8	909.4	Gda	Guanine deaminase; Catalyzes the hydrolytic deamination of guanine, producing xanthine and ammonia; Belongs to the metallo-dependent hydrolases superfamily. ATZ/TRZ family
tr|Q3TF14|Q3TF14_MOUSE	100	887.1	Ahcy	S-adenosylhomocysteine hydrolase; therefore adenosylhomocysteinase may play a key role in the control of methylations via regulation of the intracellular concentration of adenosylhomocysteine

**tr|Q3UE40|Q3UE40_MOUSE**	**99.7**	**758.1**	**Gna13**	**Guanine nucleotide-binding protein subunit alpha-13; Guanine nucleotide-binding proteins (G proteins) are involved as modulators or transducers in various transmembrane signaling systems. Activates effector molecule RhoA by binding and activating RhoGEFs (ARHGEF1/p115RhoGEF, ARHGEF11/PDZ-RhoGEF and ARHGEF12/LARG).**
**tr|D0U293|D0U293_MOUSE**	**91.2**	**277.3**	**Hbb-bt**	**Hemoglobin subunit beta-1; Involved in oxygen transport from the lung to the various peripheral tissues**
**tr|Q542G0|Q542G0_MOUSE**	**99.8**	**506.9**	**Hcls1**	**Hematopoietic lineage cell-specific protein; Substrate of the antigen receptor-coupled tyrosine kinase. Plays a role in antigen receptor signaling for both clonal expansion and deletion in lymphoid cells. May also be involved in the regulation of gene expression (By similarity)**
tr|Q1WWK3|Q1WWK3_MOUSE	97.5	195.7	Hist1h1b	Histone H1.5; Histone H1 protein binds to linker DNA between nucleosomes forming the macromolecular structure known as the chromatin fiber.
**tr|G3UVV4|G3UVV4_MOUSE**	**99.9**	**1791.2**	**Hk1**	**Hexokinase-1; Hexokinase 1; Belongs to the hexokinase family**
sp|Q3TRM8|HXK3_MOUSE	100.0	1827.4	Hk3	Hexokinase-3; Hexokinase 3; Belongs to the hexokinase family
**tr|Q3U8W9|Q3U8W9_MOUSE**	**99.7**	**953.4**	**Hnrnpr**	**Heterogeneous nuclear ribonucleoprotein R**
**sp|Q9EQ06|DHB11_MOUSE**	**100.0**	**582.4**	**Hsd17b11**	**Estradiol 17-beta-dehydrogenase 11; Can convert androstan-3-alpha,17-beta-diol (3-alpha-diol) to androsterone in vitro, suggesting that it may participate in androgen metabolism during steroidogenesis. May act by metabolizing compounds that stimulate steroid synthesis and/or by generating metabolites that inhibit it.**
tr|Q3TT11|Q3TT11_MOUSE	99.9	1472.2	Hsd17b4	Peroxisomal multifunctional enzyme type 2; Bifunctional enzyme acting on the peroxisomal beta- oxidation pathway for fatty acids.
**tr|Q71LX8|Q71LX8_MOUSE**	**100.0**	**1274.6**	**Hsp90ab1**	**Heat shock protein HSP 90-beta; Molecular chaperone that promotes the maturation, structural maintenance and proper regulation of specific target proteins involved for instance in cell cycle control and signal transduction. Undergoes a functional cycle that is linked to its ATPase activity.**
**sp|P07744|K2C4_MOUSE**	**100.0**	**748.4**	**Krt4**	**Keratin, type II cytoskeletal 4; Keratin 4**
tr|Q543N3|Q543N3_MOUSE	100.0	364.8	Lasp1	LIM and SH3 domain protein 1; Plays an important role in the regulation of dynamic actin-based, cytoskeletal activities. Agonist-dependent changes in LASP1 phosphorylation may also serve to regulate actin-associated ion transport activities, not only in the parietal cell but also in certain other F-actin-rich secretory epithelial cell types (By similarity)
tr|A0A077S2U6|A0A077S2U6_MOUSE	100.0	312.0	Lyz2	Lysozyme C-2; Lysozymes have primarily a bacteriolytic function; those in tissues and body fluids are associated with the monocyte- macrophage system and enhance the activity of immunoagents.
tr|A2AFW6|A2AFW6_MOUSE	97.0	591.3	Mtch2	Mitochondrial carrier homolog 2; The substrate transported is not yet known. Induces mitochondrial depolarization (By similarity)

**tr|L0CUZ4|L0CUZ4_MOUSE**	**83.9**	**610.5**	**Mycn**	**N-myc proto-oncogene protein; Positively regulates the transcription of MYCNOS in neuroblastoma cells**
sp|O70145|NCF2_MOUSE	100.0	1040.8	Ncf2	Neutrophil cytosol factor 2; NCF2, NCF1, and a membrane bound cytochrome b558 are required for activation of the latent NADPH oxidase (necessary for superoxide production)
tr|D3YUM1|D3YUM1_MOUSE	98.1	946.4	Ndufv1	NADH dehydrogenase [ubiquinone] flavoprotein 1, mitochondrial; Core subunit of the mitochondrial membrane respiratory chain NADH dehydrogenase (Complex I) that is believed to belong to the minimal assembly required for catalysis.
tr|Q3TF40|Q3TF40_MOUSE	100.0	651.4	Nono	Non-POU domain-containing octamer-binding protein; DNA- and RNA binding protein, involved in several nuclear processes
tr|Q3TV00|Q3TV00_MOUSE	100.0	375.6	Phb	Prohibitin; Prohibitin inhibits DNA synthesis. It has a role in regulating proliferation. As yet it is unclear if the protein or the mRNA exhibits this effect. May play a role in regulating mitochondrial respiration activity and in aging (By similarity)
**sp|P52480|KPYM_MOUSE**	**100.0**	**1047.7**	**Pkm**	**Pyruvate kinase PKM; Glycolytic enzyme that catalyzes the transfer of a phosphoryl group from phosphoenolpyruvate (PEP) to ADP, generating ATP. Stimulates POU5F1-mediated transcriptional activation (By similarity)**
**sp|Q9DBG5|PLIN3_MOUSE**	**100.0**	**709.9**	**Plin3**	**Perilipin-3; Required for the transport of mannose 6-phosphate receptors (MPR) from endosomes to the trans-Golgi network.**
tr|H3BJQ7|H3BJQ7_MOUSE	92.0	320.9	Prdx5	Peroxiredoxin-5, mitochondrial; Thiol-specific peroxidase that catalyzes the reduction of hydrogen peroxide and organic hydroperoxides to water and alcohols, respectively. Plays a role in cell protection against oxidative stress by detoxifying peroxides and as sensor of hydrogen peroxide-mediated signaling events Belongs to the peroxiredoxin family.
**sp|P28867|KPCD_MOUSE**	**96.3**	**1394.0**	**Prkcd**	**Protein kinase C delta type; Calcium-independent, phospholipid- and diacylglycerol (DAG)-dependent serine/threonine-protein kinase that plays contrasting roles in cell death and cell survival by functioning as a pro-apoptotic protein during DNA damage-induced apoptosis, but acting as an anti-apoptotic protein during cytokine receptor- initiated cell death, is required for oxygen radical production by NADPH oxidase and acts as positive or negative regulator in platelet functional responses.**
**tr|A0A1B0GS70|A0A1B0GS70_MOUSE**	**100.0**	**456.4**	**Psma1**	**Proteasome subunit alpha type-1; Component of the 20S core proteasome complex involved in the proteolytic degradation of most intracellular proteins. This complex plays numerous essential roles within the cell by associating with different regulatory particles. Associated with two 19S regulatory particles, forms the 26S proteasome and thus participates in the ATP-dependent degradation of ubiquitinated proteins.**
**tr|Q3TI61|Q3TI61_MOUSE**	**99.9**	**1655.6**	**Psmd2**	**26S proteasome non-ATPase regulatory subunit 2. This complex plays a key role in the maintenance of protein homeostasis by removing misfolded or damaged proteins, which**

				**could impair cellular functions, and by removing proteins whose functions are no longer required.**
**tr|Q4FJY5|Q4FJY5_MOUSE**	**99.4**	**659.1**	**Ptgr1**	**Prostaglandin reductase 1; Functions as 15-oxo-prostaglandin 13-reductase and acts on 15-oxo-PGE1, 15-oxo-PGE2 and 15-oxo-PGE2-alpha. Has no activity towards PGE1, PGE2 and PGE2-alpha. Catalyzes the conversion of leukotriene B4 into its biologically less active metabolite, 12- oxo-leukotriene B4. This is an initial and key step of metabolic inactivation of leukotriene B4**
tr|A0A1D5RM85|A0A1D5RM85_MOUSE	99.3	308.5	Rpl18a	Ribosomal protein L18A
tr|Q3T9U9|Q3T9U9_MOUSE	99.8	807.4	Rpl3	60S ribosomal protein L3; The L3 protein is a component of the large subunit of cytoplasmic ribosomes
tr|Q3UCH0|Q3UCH0_MOUSE	100.0	508.1	Rpl6	60S ribosomal protein L6; Component of the large ribosomal subunit
tr|Q5M9K7|Q5M9K7_MOUSE	100.0	294.3	Rps10	40S ribosomal protein S10; Component of the 40S ribosomal subunit
tr|W4VSP4|W4VSP4_MOUSE	100.0	743.8	Serpinb6c	Serine (or cysteine) peptidase inhibitor, clade B, member 6c; Belongs to the serpin family
**tr|A1L3T3|A1L3T3_MOUSE**	**99.8**	**1054.3**	**Sgsh**	**N-sulfoglucosamine sulfohydrolase (Sulfamidase), isoform CRA_b; N-sulfoglucosamine sulfohydrolase (sulfamidase)**
tr|Q3THU8|Q3THU8_MOUSE	99.7	722.6	Slc25a3	Phosphate carrier protein, mitochondrial; Transport of phosphate groups from the cytosol to mitochondrial matrix. Phosphate is cotransported with H(+). May play a role regulation of the mitochondrial permeability transition pore (mPTP) (By similarity)
**tr|G3UXX3|G3UXX3_MOUSE**	**83.6**	**404.1**	**Spr**	**Sepiapterin reductase; Catalyzes the final one or two reductions in tetra- hydrobiopterin biosynthesis to form 5,6,7,8-tetrahydrobiopterin**
**tr|D3Z1M7|D3Z1M7_MOUSE**	**98.4**	**1369.0**	**Synj1**	**Synaptojanin-1; Phosphatase that acts on various phosphoinositides, including phosphatidylinositol 4-phosphate, phosphatidylinositol (4,5)-bisphosphate and phosphatidylinositol (3,4,5)-trisphosphate (By similarity). Has a role in clathrin-mediated endocytosis (By similarity).**
**sp|Q921T2|TOIP1_MOUSE**	**87.2**	**923.7**	**Tor1aip1**	**Torsin-1A-interacting protein 1; Required for nuclear membrane integrity. Induces TOR1A and TOR1B ATPase activity and is required for their location on the nuclear membrane.**
sp|P05213|TBA1B_MOUSE	100.0	925.6	Tuba1b	Tubulin alpha-1B chain; Tubulin is the major constituent of microtubules.
**tr|B2KF55|B2KF55_MOUSE**	**60.0**	**110.9**	**Ube2v2**	**Ubiquitin-conjugating enzyme E2 variant 2; Has no ubiquitin ligase activity on its own. The UBE2V2/UBE2N heterodimer catalyzes the synthesis of non-canonical poly-ubiquitin chains that are linked through 'Lys-63'. Plays a role in the control of progress through the cell cycle and differentiation. Plays a role in the error-free DNA repair pathway and contributes to the survival of cells after DNA damage**
sp|Q9DB77|QCR2_MOUSE	100.0	860.5	Uqcrc2	Cytochrome b-c1 complex subunit 2, mitochondrial; This is a component of the ubiquinol-cytochrome c reductase complex (complex III or cytochrome b-c1 complex); Belongs to the peptidase M16 family. UQCRC2/QCR2 subfamily

tr|G3UX26|G3UX26_MOUSE	100.0	585.5	Vdac2	Voltage-dependent anion-selective channel protein 2; Forms a channel through the mitochondrial outer membrane that allows diffusion of small hydrophilic molecules.
tr|Q3TJ43|Q3TJ43_MOUSE	99.9	1535.8	Vps35	Vacuolar protein sorting-associated protein 35; Acts as component of the retromer cargo-selective complex (CSC). The CSC is believed to be the core functional component of retromer or respective retromer complex variants acting to prevent missorting of selected transmembrane cargo proteins into the lysosomal degradation pathway.
sp|Q99KC8|VMA5A_MOUSE	100.0	1558.1	Vwa5a	Von Willebrand factor A domain-containing protein 5A; May play a role in tumorigenesis as a tumor suppressor

Table S2. Exclusive or up-modulated proteins present in PECs from C57BL/6 mice infected with *T. gondii* (FC >2 compared to PECs of BALB/c infected with *T. gondii*).

QueryItem	Identity	Bitscore	PreferredName	Annotation STRING
tr|G3X9I4|G3X9I4_MOUSE	100.0	379.0	Alyref2	Aly/REF export factor 2; Export adapter involved in spliced and unspliced mRNA nuclear export. Binds mRNA which is transferred to the NXF1-NXT1 heterodimer for export (TAP/NFX1 pathway); enhances NXF1-NXT1 RNA- binding activity
**tr|Q3TUI1|Q3TUI1_MOUSE**	99.0	1296.2	Anxa6	Annexin A6; May associate with CD21. May regulate the release of Ca(2+) from intracellular stores; Belongs to the annexin family
tr|A0A1S6GWG6|A0A1S6GWG6_MOUSE	100.0	1026.5	Atp6v1b2	V-type proton ATPase subunit B, brain isoform; Non-catalytic subunit of the peripheral V1 complex of vacuolar ATPase. V-ATPase is responsible for acidifying a variety of intracellular compartments in eukaryotic cells
sp|P60766|CDC42_MOUSE	100.0	391.3	Cdc42	Cell division control protein 42 homolog; Plasma membrane-associated small GTPase which cycles between an active GTP-bound and an inactive GDP-bound state. In active state binds to a variety of effector proteins to regulate cellular responses. Involved in epithelial cell polarization processes. Plays a role in the extension and maintenance of the formation of thin, actin-rich surface projections called filopodia
**tr|Q3TNL1|Q3TNL1_MOUSE**	99.8	1069.3	G6pdx	Glucose-6-phosphate 1-dehydrogenase X; Catalyzes the rate-limiting step of the oxidative pentose-phosphate pathway, which represents a route for the dissimilation of carbohydrates besides glycolysis. The main function of this enzyme is to provide reducing power (NADPH) and pentose phosphates for fatty acid and nucleic acid synthesis; Belongs to the glucose-6-phosphate dehydrogenase family
**sp|P26443|DHE3_MOUSE**	100.0	1127.1	Glud1	Mitochondrial glutamate dehydrogenase that converts L- glutamate into alpha-ketoglutarate. Plays a key role in glutamine anaplerosis by producing alpha-ketoglutarate, an important intermediate in the tricarboxylic acid cycle. May be involved in learning and memory reactions by increasing the turnover of the excitatory neurotransmitter glutamate
**tr|Q4KL76|Q4KL76_MOUSE**	100.0	199.9	Hspe1	10 kDa heat shock protein, Co-chaperonin implicated in mitochondrial protein import and macromolecular assembly. Together with Hsp60, facilitates the correct folding of imported proteins. May also prevent misfolding and promote the refolding and proper assembly of unfolded polypeptides generated under stress conditions in the mitochondrial matrix
sp|Q497I4|KRT35_MOUSE	100.0	859.0	Krt35	Keratin, type I cuticular Ha5; Keratin 35

tr|Q3U7V7|Q3U7V7_MOUSE	99.3	282.7	Pfn1	Profilin-1; Binds to actin and affects the structure of the cytoskeleton. At high concentrations, profilin prevents the polymerization of actin, whereas it enhances it at low concentrations. By binding to PIP2, it inhibits the formation of IP3 and DG. Inhibits androgen receptor (AR) and HTT aggregation and binding of G-actin is essential for its inhibition of AR
sp|P52480|KPYM_MOUSE	100.0	1047.7	Pkm	Pyruvate kinase PKM; Glycolytic enzyme that catalyzes the transfer of a phosphoryl group from phosphoenolpyruvate (PEP) to ADP, generating ATP
tr|B1AXW5|B1AXW5_MOUSE	100.0	352.4	Prdx1	Peroxiredoxin-1; Thiol-specific peroxidase that catalyzes the reduction of hydrogen peroxide and organic hydroperoxides to water and alcohols, respectively. Plays a role in cell protection against oxidative stress by detoxifying peroxides and as sensor of hydrogen peroxide-mediated signaling events. Might participate in the signaling cascades of growth factors and tumor necrosis factor-alpha by regulating the intracellular concentrations of H(2)O(2)
sp|Q60692|PSB6_MOUSE	100.0	478.0	Psmb6	Proteasome subunit beta type-6; Component of the 20S core proteasome complex involved in the proteolytic degradation of most intracellular proteins. This complex plays numerous essential roles within the cell by associating with different regulatory particles. Associated with two 19S regulatory particles, forms the 26S proteasome and thus participates in the ATP-dependent degradation of ubiquitinated proteins. The 26S proteasome plays a key role in the maintenance of protein homeostasis by removing misfolded or damaged proteins that could impair cellular functions
tr|Q0PD67|Q0PD67_MOUSE	100.0	411.4	Rab1	Ras-related protein Rab-1A; The small GTPases Rab are key regulators of intracellular membrane trafficking, from the formation of transport vesicles to their fusion with membranes. Rabs cycle between an inactive GDP-bound form and an active GTP-bound form that is able to recruit to membranes different sets of downstream effectors directly responsible for vesicle formation, movement, tethering and fusion. RAB1A regulates vesicular protein transport from the endoplasmic reticulum (ER) to the Golgi compartment and on to the cell surface
tr|Q3ULW0|Q3ULW0_MOUSE	99.5	444.5	Ran	GTP-binding nuclear protein Ran; GTPase involved in nucleocytoplasmic transport, participating both to the import and the export from the nucleus of proteins and RNAs. Switches between a cytoplasmic GDP- and a nuclear GTP-bound state by nucleotide exchange and GTP hydrolysis. Nuclear import receptors such as importin beta bind their substrates only in the absence of GTP-bound RAN and release them upon direct interaction with GTP-bound RAN while export receptors behave in the opposite way
**tr|Q3TIQ2|Q3TIQ2_MOUSE**	99.4	330.9	Rpl12	60S ribosomal protein L12; Binds directly to 26S ribosomal RNA

sp|P54116|STOM_MOUSE	100.0	529.6	Stom	Erythrocyte band 7 integral membrane protein; Regulates ion channel activity and transmembrane ion transport. Regulates ASIC2 and ASIC3 channel activity; Belongs to the band 7/mec-2 family
tr|A0A0A6YW67|A0A0A6YW67_MOUSE	100.0	153.7	Ubb	Polyubiquitin-B; Exists either covalently attached to another protein, or free (unanchored). When covalently bound, it is conjugated to target proteins via an isopeptide bond either as a monomer (monoubiquitin), a polymer linked via different Lys residues of the ubiquitin (polyubiquitin chains) or a linear polymer linked via the initiator Met of the ubiquitin (linear polyubiquitin chains)
tr|A2A5N2|A2A5N2_MOUSE	100.0	468.8	Ywhab	14-3-3 protein beta/alpha; Adapter protein implicated in the regulation of a large spectrum of both general and specialized signaling pathways. Binds to a large number of partners, usually by recognition of a phosphoserine or phosphothreonine motif. Binding generally results in the modulation of the activity of the binding partner. Negative regulator of osteogenesis. Blocks the nuclear translocation of the phosphorylated form (by AKT1) of SRPK2 and antagonizes its stimulatory effect on cyclin D1 expression resulting in blockage of neuronal apoptosis elicited by SRPK2
sp|P68510|1433F_MOUSE	100.0	474.6	Ywhah	14-3-3 protein eta; Adapter protein implicated in the regulation of a large spectrum of both general and specialized signaling pathways. Binds to a large number of partners, usually by recognition of a phosphoserine or phosphothreonine motif. Negatively regulates the kinase activity of PDPK1

Supplementary Table S3. Proteins down-modulated (FC < 1.0) exclusively found in PECs from C57BL/6 mice treated with rSAG2A.

QueryItem	Identity	Bitscore	PreferredName	annotation
**tr|Q8BJT5|Q8BJT5_MOUSE**	**99.4**	**342.0**	**Acadm**	**Medium-chain specific acyl-CoA dehydrogenase, mitochondrial; Acyl-CoA dehydrogenase specific for acyl chain lengths of 4 to 16 that catalyzes the initial step of fatty acid beta-oxidation.**
sp|Q9CQI6|COTL1_MOUSE	100.0	286.2	Cotl1	Coactosin-like protein; Binds to F-actin in a calcium-independent manner. Acts as a chaperone for ALOX5 (5LO), influencing both its stability and activity in leukotrienes synthesis (By similarity)
sp|Q9CZU6|CISY_MOUSE	100.0	941.4	Cs	Citrate synthase, mitochondrial; Citrate synthase
tr|Q3TVU9|Q3TVU9_MOUSE	99.7	634.8	Dhrs1	Dehydrogenase/reductase (SDR family) member 1; Belongs to the short-chain dehydrogenases/reductases (SDR) family
tr|A0A0N4SUX5|A0A0N4SUX5_MOUSE	89.9	770.0	Eno2	Gamma-enolase; Has neurotrophic and neuroprotective properties on a broad spectrum of central nervous system (CNS) neurons.
tr|H3BJL6|H3BJL6_MOUSE	100.0	545.8	Esd	S-formylglutathione hydrolase; Serine hydrolase involved in the detoxification of formaldehyde
tr|Q3TJH1|Q3TJH1_MOUSE	99.7	704.5	Gnai3	Guanine nucleotide-binding protein G(k) subunit alpha; Heterotrimeric guanine nucleotide-binding proteins (G proteins) function as transducers downstream of G protein-coupled receptors (GPCRs) in numerous signaling cascades.
**tr|Q3TGJ9|Q3TGJ9_MOUSE**	**99.7**	**1484.5**	**Gsn**	**Gelsolin; Calcium-regulated, actin-modulating protein that binds to the plus (or barbed) ends of actin monomers or filaments, preventing monomer exchange (end-blocking or capping). It can promote the assembly of monomers into filaments (nucleation) as well as sever filaments already formed. Plays a role in ciliogenesis**
sp|Q6ZWY9|H2B1C_MOUSE	100.0	193.7	Hist1h2bg	Histone H2B type 1-C/E/G; Core component of nucleosome. Nucleosomes wrap and compact DNA into chromatin, limiting DNA accessibility to the cellular machineries which require DNA as a template.
sp|Q3UQ44|IQGA2_MOUSE	100.0	2852.4	Iqgap2	Ras GTPase-activating-like protein IQGAP2; Binds to activated CDC42 and RAC1 but does not seem to stimulate their GTPase activity. Associates with calmodulin
**tr|Q3V471|Q3V471_MOUSE**	**100.0**	**274.6**	**Lgals3**	**Galectin-3; Galactose-specific lectin which binds IgE. Together with DMBT1, required for terminal differentiation of columnar epithelial cells during early embryogenesis. Involved in acute inflammatory responses including neutrophil activation and adhesion, chemoattraction of monocytes macrophages, opsonization of apoptotic neutrophils**
sp|P48678|LMNA_MOUSE	100.0	814.3	Lmna	Prelamin-A/C. Plays an important role in nuclear assembly, chromatin organization, nuclear membrane and telomere dynamics.
**tr|O35558|O35558_MOUSE**	**97.1**	**553.5**	**Mapk1**	**Mitogen-activated protein kinase 1**
sp|P08249|MDHM_MOUSE	100.0	664.5	Mdh2	Malate dehydrogenase 2, NAD (mitochondrial)

sp|P60335|PCBP1_MOUSE	100.0	698.0	Pcbp1	Poly(rC)-binding protein 1; Single-stranded nucleic acid binding protein that binds preferentially to oligo dC
**tr|Q003I5|Q003I5_MOUSE**	**99.7**	**1478.8**	**Pdgfrb**	**Platelet-derived growth factor receptor beta; Tyrosine-protein kinase that acts as cell-surface receptor for homodimeric PDGFB and PDGFD and for heterodimers formed by PDGFA and PDGFB, and plays an essential role in the regulation of embryonic development, cell proliferation, survival, differentiation, chemotaxis and migration.**
sp|P09411|PGK1_MOUSE	100.0	832.0	Pgk1-rs7	Phosphoglycerate kinase 1; In addition to its role as a glycolytic enzyme, it seems that PGK-1 acts as a polymerase alpha cofactor protein (primer recognition protein)
**tr|Q3TLP8|Q3TLP8_MOUSE**	**91.0**	**388.3**	**Rac1**	**Ras-related C3 botulinum toxin substrate 1; Plasma membrane-associated small GTPase which cycles between active GTP-bound and inactive GDP-bound states. In its active state, binds to a variety of effector proteins to regulate cellular responses such as secretory processes, phagocytosis of apoptotic cells.**
tr|Q5M9L1|Q5M9L1_MOUSE	98.1	197.2	Rpl36	60S ribosomal protein L36; Component of the large ribosomal subunit
tr|Q4FZE6|Q4FZE6_MOUSE	100.0	371.3	Rps7	40S ribosomal protein S7; Required for rRNA maturation; Belongs to the eukaryotic ribosomal protein eS7 family
tr|Q545I9|Q545I9_MOUSE	100.0	176.0	S100a6	Protein S100-A6; May function as calcium sensor and modulator, contributing to cellular calcium signaling. May function by interacting with other proteins, such as TPR-containing proteins, and indirectly play a role in many physiological processes such as the reorganization of the actin cytoskeleton and in cell motility. Binds 2 calcium ions.
tr|A0A1B0GR11|A0A1B0GR11_MOUSE	88.2	642.9	Taldo1	Transaldolase; Transaldolase is important for the balance of metabolites in the pentose-phosphate pathway
sp|Q6IRU2|TPM4_MOUSE	100.0	89.7	Tpm4	Tropomyosin alpha-4 chain; Binds to actin filaments in muscle and non-muscle cells. Plays a central role, in association with the troponin complex, in the calcium dependent regulation of vertebrate striated muscle contraction.
sp|Q64727|VINC_MOUSE	100.0	1862.4	Vcl	Vinculin; Actin filament (F-actin)-binding protein involved in cell-matrix adhesion and cell-cell adhesion. May also play important roles in cell morphology and locomotion (By similarity); Belongs to the vinculin/alpha-catenin family
tr|Q3THL7|Q3THL7_MOUSE	99.6	573.9	Vdac1	Voltage-dependent anion-selective channel protein 1; Forms a channel through the mitochondrial outer membrane and also the plasma membrane. The channel at the outer mitochondrial membrane allows diffusion of small hydrophilic molecules; in the plasma membrane it is involved in cell volume regulation and apoptosis.

Supplementary Table S4. Proteins down-modulated (FC < 1.0) exclusively found in PECs from C57BL/6 mice infected with *T. gondii*.

QueryItem	Identity	Bitscore	PreferredName	Annotation STRING
**tr|Q3TFK4|Q3TFK4_MOUSE**	99.7	613.6	Anxa3	Annexin A3; Inhibitor of phospholipase A2, also possesses anti- coagulant properties
tr|Q3TCE7|Q3TCE7_MOUSE	99.7	639.8	Arpc1b	Actin-related protein 2/3 complex subunit 1B; Functions as component of the Arp2/3 complex which is involved in regulation of actin polymerization and together with an activating nucleation-promoting factor (NPF) mediates the formation of branched actin networks
sp|P56480|ATPB_MOUSE	100.0	1012.7	Atp5b	ATP synthase subunit beta, mitochondrial. F-type ATPases consist of two structural domains, F(1) - containing the extramembraneous catalytic core, and F(0) - containing the membrane proton channel, linked together by a central stalk and a peripheral stalk
sp|P50516|VATA_MOUSE	100.0	1232.2	Atp6v1a	V-type proton ATPase catalytic subunit A; V-ATPase vacuolar ATPase is responsible for acidifying a variety of intracellular compartments in eukaryotic cells. In aerobic conditions, involved in intracellular iron homeostasis, thus triggering the activity of Fe(2+) prolyl hydroxylase (PHD) enzymes, and leading to HIF1A hydroxylation and subsequent proteasomal degradation
tr|A0A0A6YWP6|A0A0A6YWP6_MOUSE	91.1	845.5	Atp6v1h	V-type proton ATPase subunit H; Subunit of the peripheral V1 complex of vacuolar ATPase. Vacuolar ATPase is responsible for acidifying a variety of intracellular compartments in eukaryotic cells, thus providing most of the energy required for transport processes in the vacuolar system. Involved in the endocytosis mediated by clathrin-coated pits, required for the formation of endosomes
sp|Q9CY64|BIEA_MOUSE	100.0	576.6	Blvra	Biliverdin reductase A; Reduces the gamma-methene bridge of the open tetrapyrrole, biliverdin IX alpha, to bilirubin with the concomitant oxidation of a NADH or NADPH cofactor
tr|Q5SXR6|Q5SXR6_MOUSE	99.7	3333.1	Cltc	Clathrin heavy chain 1; Clathrin is the major protein of the polyhedral coat of coated pits and vesicles. Acts as component of the TACC3/ch- TOG/clathrin complex proposed to contribute to stabilization of kinetochore fibers of the mitotic spindle by acting as inter-microtubule bridge. The TACC3/ch-TOG/clathrin complex is required for the maintenance of kinetochore fiber tension. Plays a role in early autophagosome formation
sp|Q9D1A2|CNDP2_MOUSE	100.0	955.3	Cndp2	Cytosolic non-specific dipeptidase; Hydrolyzes a variety of dipeptides including L-carnosine but has a strong preference for Cys-Gly. Catalyzes the production of N-lactoyl-amino acids from lactate and amino acids by reverse proteolysis

sp|Q9D2G2|ODO2_MOUSE	100.0	780.0	Dlst	Dihydrolipoamide S-succinyltransferase (E2 component of 2-oxo-glutarate complex); The 2-oxoglutarate dehydrogenase complex catalyzes the overall conversion of 2-oxoglutarate to succinyl-CoA and CO(2)
sp|P57759|ERP29_MOUSE	100.0	503.8	Erp29	Endoplasmic reticulum resident protein 29; Does not seem to be a disulfide isomerase. Plays an important role in the processing of secretory proteins within the endoplasmic reticulum (ER), possibly by participating in the folding of proteins in the ER
tr|Q3TEE6|Q3TEE6_MOUSE	100.0	1315.1	Fermt3	Fermitin family homolog 3; Plays a central role in cell adhesion in hematopoietic cells. Acts by activating the integrin beta-1-3 (ITGB1, ITGB2 and ITGB3). Required for integrin-mediated platelet adhesion and leukocyte adhesion to endothelial cells. Required for activation of integrin beta- 2 (ITGB2) in polymorphonuclear granulocytes (PMNs)
sp|P09528|FRIH_MOUSE	100.0	372.9	Fth1	Ferritin heavy chain; Stores iron in a soluble, non-toxic, readily available form. Important for iron homeostasis. Has ferroxidase activity. Iron is taken up in the ferrous form and deposited as ferric hydroxides after oxidation. Also plays a role in delivery of iron to cells
**tr|A0A0R4J0X7|A0A0R4J0X7_MOUSE**	100.0	692.2	Gapdhs	Glyceraldehyde-3-phosphate dehydrogenase, testis-specific; May play an important role in regulating the switch between different pathways for energy production during spermiogenesis and in the spermatozoon
sp|P08752|GNAI2_MOUSE	100.0	709.5	Gnai2	Guanine nucleotide-binding proteins (G proteins) are involved as modulators or transducers in various transmembrane signaling systems. The G(i) proteins are involved in hormonal regulation of adenylate cyclase: they inhibit the cyclase in response to beta-adrenergic stimuli.
tr|K7W4D1|K7W4D1_MOUSE	87.5	519.6	H2-K1	H-2 class I histocompatibility antigen, K-B alpha chain; Involved in the presentation of foreign antigens to the immune system; Belongs to the MHC class I family
tr|B7ZP22|B7ZP22_MOUSE	99.7	388.3	Hnrnpa2b1	Heterogeneous nuclear ribonucleoprotein (hnRNP) that associates with nascent pre-mRNAs, packaging them into hnRNP particles. The hnRNP particle arrangement on nascent hnRNA is non-random and sequence-dependent and serves to condense and stabilize the transcripts and minimize tangling and knotting
**tr|Q3TKA2|Q3TKA2_MOUSE**	99.7	1266.9	Hsp90aa1	Heat shock protein HSP 90-alpha; Molecular chaperone that promotes the maturation, structural maintenance and proper regulation of specific target proteins involved for instance in cell cycle control and signal transduction. Undergoes a functional cycle that is linked to its ATPase activity which is essential for its chaperone activity. This cycle probably induces conformational changes in the client proteins, thereby causing their activation

tr|Q6ZQK2|Q6ZQK2_MOUSE	99.9	2993.8	Iqgap1	Ras GTPase-activating-like protein IQGAP1; Binds to activated CDC42 but does not stimulate its GTPase activity. It associates with calmodulin. Could serve as an assembly scaffold for the organization of a multimolecular complex that would interface incoming signals to the reorganization of the actin cytoskeleton at the plasma membrane
tr|E9Q390|E9Q390_MOUSE	99.8	4164.8	Myof	Myoferlin; Calcium/phospholipid-binding protein that plays a role in the plasmalemma repair mechanism of endothelial cells that permits rapid resealing of membranes disrupted by mechanical stress. Involved in endocytic recycling. Implicated in VEGF signal transduction by regulating the levels of the receptor KDR
**tr|Q3UAG2|Q3UAG2_MOUSE**	99.8	975.7	Pgd	6-phosphogluconate dehydrogenase, decarboxylating; Catalyzes the oxidative decarboxylation of 6- phosphogluconate to ribulose 5-phosphate and CO(2), with concomitant reduction of NADP to NADPH
sp|Q9QXS1|PLEC_MOUSE	99.9	4615.4	Plec	Plectin; Interlinks intermediate filaments with microtubules and microfilaments and anchors intermediate filaments to desmosomes or hemidesmosomes.
tr|Q05DV1|Q05DV1_MOUSE	100.0	1223.0	Por	NADPH--cytochrome P450 reductase; This enzyme is required for electron transfer from NADP to cytochrome P450 in microsomes. It can also provide electron transfer to heme oxygenase and cytochrome B5
tr|Q9DCY1|Q9DCY1_MOUSE	100.0	434.5	Ppib	Peptidyl-prolyl cis-trans isomerase B; PPIases accelerate the folding of proteins. It catalyzes the cis-trans isomerization of proline imidic peptide bonds in oligopeptides
tr|Q8C2E1|Q8C2E1_MOUSE	100.0	391.7	Ppp2r1a	Serine/threonine-protein phosphatase 2A 65 kDa regulatory subunit A alpha isoform. Required for proper chromosome segregation and for centromeric localization of SGO1 in mitosis
tr|G3X9V0|G3X9V0_MOUSE	100.0	440.7	Psme2	Proteasome activator complex subunit 2; Implicated in immunoproteasome assembly and required for efficient antigen processing. The PA28 activator complex enhances the generation of class I binding peptides by altering the cleavage pattern of the proteasome
tr|Q3TE70|Q3TE70_MOUSE	99.8	1181.8	Ptpn6	Tyrosine-protein phosphatase non-receptor type 6; Modulates signaling by tyrosine phosphorylated cell surface receptors such as KIT and the EGF receptor/EGFR. The SH2 regions may interact with other cellular components to modulate its own phosphatase activity against interacting substrates. Together with MTUS1, induces UBE2V2 expression upon angiotensin II stimulation. Plays a key role in hematopoiesis; Belongs to the protein-tyrosine phosphatase family. Non-receptor class 2 subfamily

tr|A0A0G2JDL9|A0A0G2JDL9_MOUSE	64.1	209.9	Rap1a	Ras-related protein Rap-1A; Induces morphological reversion of a cell line transformed by a Ras oncogene. Counteracts the mitogenic function of Ras, at least partly because it can interact with Ras GAPs and RAF in a competitive manner. Together with ITGB1BP1, regulates KRIT1 localization to microtubules and membranes. Plays a role in nerve growth factor (NGF)-induced neurite outgrowth
**tr|Q4VA28|Q4VA28_MOUSE**	**99.4**	**321.2**	**Rpl21**	**60S ribosomal protein L21; Component of the large ribosomal subunit**
**tr|Q3TKR5|Q3TKR5_MOUSE**	**99.7**	**577.0**	**Rpl5**	**60S ribosomal protein L5; Component of the ribosome, a large ribonucleoprotein complex responsible for the synthesis of proteins in the cell. The small ribosomal subunit (SSU) binds messenger RNAs (mRNAs) and translates the encoded message by selecting cognate aminoacyl- transfer RNA (tRNA) molecules**
**tr|A0A1W2P7A1|A0A1W2P7A1_MOUSE**	**100.0**	**260.4**	**Rps12-ps3**	**Ribosomal protein S12, pseudogene 3**
**tr|Q3UK56|Q3UK56_MOUSE**	99.2	471.5	Rps3	40S ribosomal protein S3; Involved in translation as a component of the 40S small ribosomal subunit (By similarity). Has endonuclease activity and plays a role in repair of damaged DNA. Cleaves phosphodiester bonds of DNAs containing altered bases with broad specificity and cleaves supercoiled DNA more efficiently than relaxed DNA (By similarity). Displays high binding affinity for 7,8-dihydro-8-oxoguanine (8-oxoG), a common DNA lesion caused by reactive oxygen species (ROS) (By similarity)
sp|Q91W10|S39A8_MOUSE	100.0	916.8	Slc39a8	Zinc transporter ZIP8; Acts as a manganese and zinc influx transporter. Plays a role in manganese reabsorption in the proximal tubule of the kidney and in manganese uptake into the brain
tr|Q3TGS7|Q3TGS7_MOUSE	100.0	335.5	Snx12	Sorting nexin-12; May be involved in several stages of intracellular trafficking
sp|P08228|SODC_MOUSE	100.0	315.5	Sod1	Superoxide dismutase [Cu-Zn]; Destroys radicals which are normally produced within the cells and which are toxic to biological systems; Belongs to the Cu-Zn superoxide dismutase family
sp|P11983|TCPA_MOUSE	100.0	1067.0	Tcp1	T-complex protein 1 subunit alpha; Molecular chaperone; assists the folding of proteins upon ATP hydrolysis. As part of the BBS/CCT complex may play a role in the assembly of BBSome, a complex involved in ciliogenesis regulating transports vesicles to the cilia.
sp|Q3TW96|UAP1L_MOUSE	100.0	1027.3	Uap1l1	UDP-N-acetylhexosamine pyrophosphorylase-like protein 1; UDP-N-acteylglucosamine pyrophosphorylase 1-like 1
